# Control of Endogenous Auxin Levels in Plant Root Development

**DOI:** 10.3390/ijms18122587

**Published:** 2017-12-01

**Authors:** Damilola Olatunji, Danny Geelen, Inge Verstraeten

**Affiliations:** 1 Department of Plant Production, Faculty of Bioscience Engineering, Ghent University, Coupure Links 653, 9000 Gent, Belgium; Damilola.olatunji@ugent.be (D.O.); danny.geelen@ugent.be (D.G.); 2 Institute of Science and Technology Austria, Am Campus 1, 3400 Klosterneuburg, Austria

**Keywords:** auxin biosynthesis, root development, conjugation, metabolism

## Abstract

In this review, we summarize the different biosynthesis-related pathways that contribute to the regulation of endogenous auxin in plants. We demonstrate that all known genes involved in auxin biosynthesis also have a role in root formation, from the initiation of a root meristem during embryogenesis to the generation of a functional root system with a primary root, secondary lateral root branches and adventitious roots. Furthermore, the versatile adaptation of root development in response to environmental challenges is mediated by both local and distant control of auxin biosynthesis. In conclusion, auxin homeostasis mediated by spatial and temporal regulation of auxin biosynthesis plays a central role in determining root architecture.

## 1. Introduction

Flowering plants have evolved a high level of developmental and morphological plasticity to accommodate adaptive growth in response to diverse environmental stimuli [1]. Unlike organ growth in animals, most plant organs are formed post-embryonically and emerge from inherent structures known as meristems [2]. Throughout the plant’s life span, meristems remain present and provide the basis for the plant’s developmental plasticity [3,4]. Initially at the embryonic state, two types of meristems occur: the shoot apical meristem (SAM), which gives rise to above ground tissues and organs and the root apical meristem (RAM), which establishes the root architecture. The root organ appears to have evolved secondary to the shoot allowing plants to invade land and facilitates the uptake of water for surviving the dry conditions [5]. The diversification of plants with the generation of varied plant forms, from tiny mosses to giant sequoia trees and from annual weeds to long-living perennials, was only possible after the emergence of a root system. Following the initiation of roots, features such as secondary growth, gravitropic responses and the development of lateral root branches are important additions that improved the function of the root system and required more complex cellular communication [5,6]. In particular, the roots of flowering plants have evolved highly specialized functions while maintaining developmental and morphological plasticity in order to cope with diverse environmental stimuli [1].

Plant roots explore the soil environment in search for nutrients and water required for growth, development and reproduction while containing a vascular system to provide organs at a distance with these necessary nutrients, water and hormones [7,8]. Generally, the root system consists of two principal root-types: the primary root (PR), which is formed embryonically [9] and secondary roots, which form post-embryonically. These secondary roots encompass both lateral roots (LR), which develop as branches of the primary root and adventitious roots (AR), which develop on non-root tissue such as the hypocotyl, stems and leaves [10,11]. The root architecture of monocots and dicots is highly distinct. Dicots have a tap-root system with a central primary root and lateral roots branching of, while monocots have a fibrous root system consisting of mainly crown or adventitious roots [12].

The plant growth regulator auxin is involved in basically all cellular processes including cell-division and -expansion with implications for all plant developmental processes [13]. Already at an early stage in embryogenesis, auxin determines cell division, tissue patterning and organ development. Likewise, at later developmental stages, auxin is involved in physiological growth responses such as gravitropism and shoot branching [6,14,15,16]. Since cell-division and -expansion requires to be spatiotemporally regulated, an appropriate auxin distribution across the tissue is highly important to coordinate growth and tissue development. Auxin homeostasis is assured not only by appropriate transport but also involves de novo biosynthesis, conjugation, storage, oxidation and catabolism, all of which need to take place in a coordinate fashion [17,18,19,20,21,22].

As an omnipotent regulator of root development, auxin controls the development and architecture of the root system [6,14]. In response to environmental cues, root growth is adapted through the modulation of endogenous auxin levels [23,24]. The establishment of auxin gradients in the root requires the interplay of local auxin biosynthesis [25,26,27,28], transport [29,30,31,32,33], perception and signaling [34,35,36]. In this review, we focus on the plant’s regulation of the endogenous auxin concentrations by biosynthesis, meta- and catabolism and conjugation. We provide information on how these processes are important for root development, encompassing the embryonic root, the PR and the secondary LR and AR. In the next paragraph, we briefly describe the molecular aspects of auxin perception (more can be found in e.g. References [37,38]). For reviews regarding the transport of auxin controlling root development see [16,39].

Auxin signals are perceived intracellularly by a family of *AUXIN SIGNALING F-BOX (AFB)* receptors of which *(TIR1) TRANSPORT INHIBITOR RESPONSE1* is the founder. These F-box proteins are the substrate-binding subunit of SCF-type ubiquitin protein ligase complexes, named after their subunits Skp1, Cullin and an F-box [38]. In the presence of auxin, the SCF^TIR1/*AFB*^-complex is brought in close proximity to Aux/IAA (AUXIN/INDOLE-3-ACETIC ACID) proteins, which allows their ubiquitinylation and consequent targeting for proteasome degradation [40]. Normally, the Aux/IAA proteins act as transcriptional repressors by binding to Auxin Response Factor (*ARF*) transcription factors [41]. The removal of Aux/IAAs upon auxin perception hence results in the activation of *ARF*s, which on their turn activate the auxin responses. The model species *Arabidopsis thaliana* contains 6 *AFB*s [42,43], 29 AUX/IAAs [44] and 23 *ARF*s [45]. Different *TIR1*/*AFB*-Aux/IAA combinations may contribute to different transcriptional responses, depending on their presence in certain tissues or the physiological status of the plants. However, this field and the resulting variety of transcriptional responses is still rather ambiguous and predicting the function of a certain *TIR1*/*AFB*-Aux/IAA pair will require much more research [46]. Besides multiple combinations between receptors and Aux/IAAs, also the plenitude of *ARF*s might contribute to the specificity of the auxin response [38]. Some of the cellular responses to auxin take place at the cell surface. A candidate receptor of auxin at the plasma membrane is AUXIN BINDING PROTEIN 1 (ABP1) [34,36,47]. ABP1 is found at the plasma membrane and in the endoplasmatic reticulum and is shown to act via non-transcriptional processes [38,48]. Recently however, the contribution of ABP1 to auxin signaling and plant development has been questioned and we are awaiting its re-examination as a regulator of auxin perception [49,50].

The differential affinity of ABP1 and *TIR1* for synthetic analogues of auxin suggests that different endogenous auxin analogues may contribute to a higher complexity of auxin signaling, possibly by separate ABP1 and *TIR1*/*AFB* modules [51]. So far four endogenous molecules—indole-3-acetic acid (IAA), indole-3-butyric acid (IBA), 4-chloroindole-3-acetic acid (4-Cl-IAA) and phenylacetic acid (PAA)—exhibit auxinic activity in plants [48,52,53,54]. IAA is most-studied and omnipresent in plants [17]. In planta, IAA is likely converted to IBA in a reversible reaction catalyzed by IBA synthetase but IBA could also serve as an IAA-precursor or -conjugate. Reversible β-oxidation of IBA to generate active IAA is a slow process and this slow release of active auxin could be the reason for the long-lasting function of IBA in rooting powders. Candidates for IBA to IAA conversion are four peroxisomal enzymes, three INDOLE- 3-BUTYRIC ACID RESPONSE (IBR) isozymes and one ENOYL–COA HYDRATASE2 (ECH2) [55,56,57]. IBA has been identified in a number of plant species such as maize (*Zea mays*), pea (*Pisum sativum*) and *Arabidopsis thaliana* but its inherent auxin-activity is still uncertain. Several studies provide evidence for a role of endogenous IBA in root development but a direct role in auxin signaling remains elusive [19,55,58,59]. The endogenous auxin-analogue PAA can bind to ABP1 but inhibits carrier-mediated auxin transport [48,60]. 4-Cl-IAA on the other hand has strong auxinic effects but also modulates processes that IAA does not affect and can be found exclusively in leguminous species [48,61]. Active auxins together with conjugated auxins constitute the auxin pool in a plant. In particular exogenous application of auxin leads to an increase of auxin-conjugating enzymes whereas conjugate hydrolases are activated to release stored inactive auxin in conjunction with initiation of growth such as during germination [62,63]. Besides the naturally occurring auxins, there are multiple synthetic auxin-derivatives, many of which were developed because of herbicide activity such as for example 2,4-dichlorophenoxyacetic acid (2,4-d), 1-naphthaleneacetic acid (NAA), dicamba and picloram [64].

## 2. Auxin Biosynthesis

Dissecting the auxin biosynthetic pathway has been proven difficult, largely because several auxin biosynthesis routes exist and the enzymes involved belong to gene families that show redundant functions [65,66,67]. Moreover, de novo auxin biosynthesis is not strictly confined to a specific tissue, although auxin is primarily synthesized in young, not fully expanded leaves [68,69]. Even though biosynthesis is the highest in the younger leaves, the largest auxin pools are measured in the root and in the expanding tissues where auxin is essential for the organ to grow. This underscores the importance of efficient transport between biosynthesis site and action site [68,70,71]. Other parts of the plant maintain the capacity to synthesize auxin as evidenced by the local production upon wounding or other local stimuli [25,69]. Below, we summarize recent findings on de novo auxin biosynthesis, which help to regulate the outcome of auxin homeostasis on root development.

The first enzymes identified to be engaged in the biosynthesis of auxin were from bacterial origin. The genes *iaaM* and *iaaH* were discovered in *Pseudomonas* and *Agrobacterium* and these enzymes respectively encode tryptophan-2-monooxygenase, which catalyzes the conversion of tryptophan to indole-3-acetamide (IAM) and a hydrolase that releases IAA [66,72]. Klee et al. [73] engineered petunia plants to express *iaaM* and this resulted in approximately a 10-fold increase in endogenous IAA-levels. Plants overproducing IAA showed typical auxin phenotypes such as excessive xylem and phloem development and bigger epidermal and palisade cells, which together with an extended intercellular space give rise to thicker leaves. Other bacterial IAA-biosynthetic pathways were soon thereafter discovered and for example, IPyA decarboxylase (IPDC) was identified to synthesize indole-3-pyruvic acid (IPyA) as an intermediate of auxin biosynthesis [74,75,76]. Orthologous of *iaaM* and *iaaH* have not been found in plants whereas the IPyA-mediated IAA-biosynthetic pathway was shown to occur in plants [25,67].

The predominant auxin biosynthesis route uses tryptophan as the main precursor. This was concluded in feeding experiments with labelled tryptophan (Trp), resulting in the production of labelled IAA, which indicated that plants use Trp as a precursor and contain the enzymes to convert Trp into IAA [77,78]. Tryptophan is synthesized from chorismate—the final product of the shikimate pathway—via intermediate indole-3-glycerol phosphate (IGP) in the chloroplasts [79]. In *Arabidopsis thaliana*, *ASA1* and *ASA2* encode the subunits of anthranilate synthase (AS), the enzyme necessary for the first step of tryptophan synthesis. Indole-3-glycerol phosphate synthase (IGS) is then responsible for the generation of IGP, a key intermediate in auxin biosynthesis because it can either form tryptophan or be used as a precursor for the tryptophan-independent auxin biosynthetic pathway [80]. Further conversion to tryptophan occurs through a heterotetrameric a_2_b_2_ complex consisting of Trp synthase a (TSA1) and Trp synthase b (TSB, in Arabidopsis there are 2 closely related versions of this gene: *TSB1* and *TSB2*) [81]. A tryptophan-deficient Arabidopsis mutant, defective in anthranilate phosphoribosyl-transferase, accumulates anthranilic acid derivatives and these interfere with IAA-biosynthesis [82]. The observed morphological defects, including slow growth, small crinkled leaves and reduced apical dominance, also occur in mutants deficient in auxin signaling such as the auxin-resistant *axr1*, a gene encoding a subunit of the RUB1-activating enzymes regulating SCF-complex-mediated protein degradation [83]. The four most well-characterized auxin pathways use Trp as a precursor and are discussed below. Even though one of these pathways—the IPyA pathway—is considered the main route for auxin biosynthesis, the other routes act in their own way, in parallel or in a developmentally/environmentally-regulated manner.

### 2.1. The Indole-3-Pyruvic Acid (IPyA) Pathway

**Figure 1:**
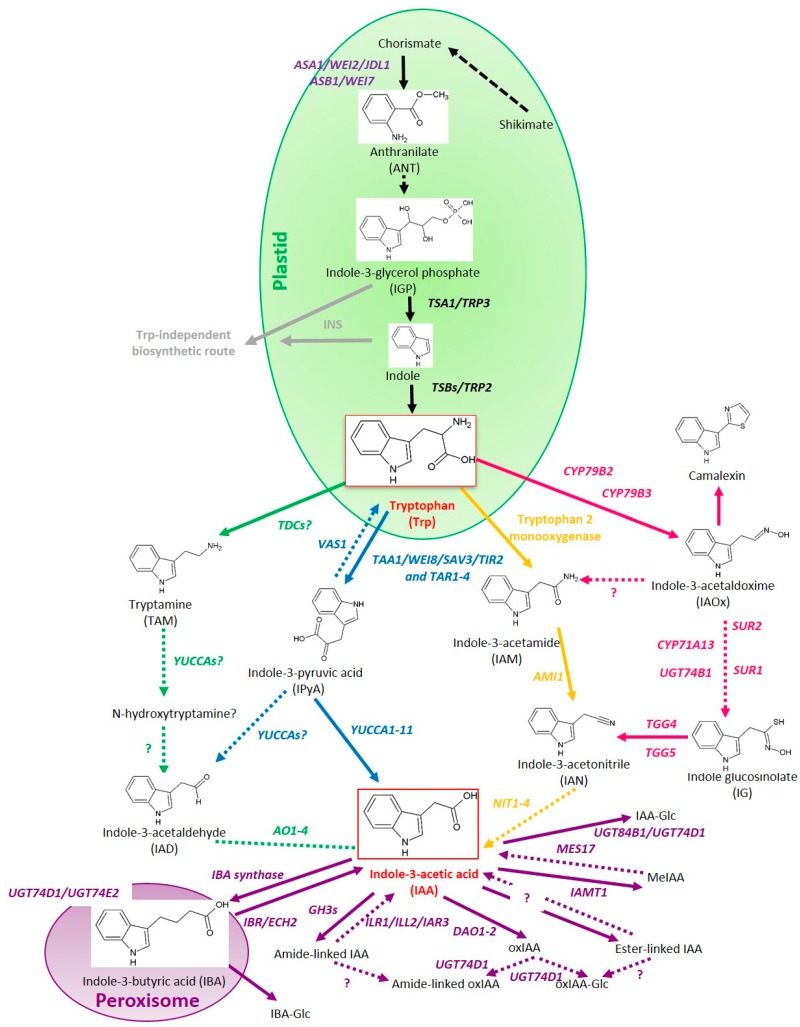
Auxin biosynthetic pathways in *Arabidopsis thaliana*. The IPyA pathway is shown in blue, the TAM pathway in green, the IAOx pathway in pink, the IAM pathway in yellow, the Trp-independent pathway in gray and the proposed metabolic pathways in purple. Enzymatic reactions without a known catalyzing enzyme are shown by dashed arrows. AO: aldehyde oxidases; IAA: 3-indole acetic acid; IAD: indole-3-acetaldehyde; IAM: indole-3-acetamide; IAN: indole-3-acetonitrile; IAOx: indole-3-acetaldoxime; IBA: indole-3-butyric acid; IG: indole glucosinolates; *IGS*: Indole-3-glycerol phosphate synthase; IPyA: indole-3-pyruvic acid; *TAA1: TRYPTOPHAN AMINOTRANSFERASE OF ARABIDOPSIS 1*; *TAR1-4: TRYPTOPHAN AMINOTRANSFERASE RELATED 1-4*; TAM: tryptamine; TRP: tryptophan; *TSA1*: Trp synthase a; *TSB*: Trp synthase b; *YUC1-11*: *yucca* genes encoding flavin-containing monooxygenases.

The existence of an IPyA auxin biosynthesis pathway was predicted based on the detection of certain intermediates and radiolabeling of tryptophan-derived metabolites [84]. The instability of IPyA at room temperature and its occurrence in two tautomer forms hampered the identification of enzymes involved [85]. The IPyA auxin biosynthesis enzymes were discovered only recently via genetic screens directed to identify regulators of organ development, ethylene response and shade avoidance. Defects in the corresponding genes lead to poor adaptation to low light conditions indicating the importance of this route of auxin biosynthesis in developmental plasticity. The IPyA pathway is a two-step reaction involving tryptophan aminotransferases (*TAA1* and *TAR*s) and Flavin-containing monooxygenases of the *YUCCA* family (*YUC1-11*) [86,87,88,89] (Blue Pathway in Figure 1). The Trp aminotransferase required for the first step were discovered by screening for ethylene insensitivity in roots, identifying *wei8* (*weak ethylene insensitive 8*) [25] and by screening for lack of a shade avoidance response, identifying *sav3* (*shade avoidance 3*) [67]. *Wei8* and *sav3* encode an aminotransferase that was renamed *TAA1* (*TRYPTOPHAN AMINOTRANSFERASE OF ARABIDOPSIS 1*). *TAA1* catalyzes the transfer of the amino group from Trp to pyruvate or to α-ketoglutarate generating IPyA and the amino acids alanine or glutamine. Both the *sav3* and *wei8* mutants contain approximately 60% of the wild-type IAA-levels and show a strong impairment in dark induced hypocotyl elongation [67]. Another short hypocotyl mutant *TRANSPORT INHIBITOR RESPONSE2* (*TIR2*) was shown to be allelic to *TAA1* [26]. Overexpression of *TAA1* causes modest auxin phenotypes indicating that this enzyme does not catalyze a rate-limiting biosynthesis step. The Arabidopsis *TAA1* belongs to a small gene family that includes the homologues *TAR1* to *4* (*TRYPTOPHAN AMINOTRANSFERASE RELATED 1-4*), which have overlapping functions [25]. Pea contains three *TAA1* homologs: *PsTAR1-3,* which were shown to synthesize *4*-Cl-IAA in addition to the canonical IAA [90].

Tryptophan feeding experiments identified a group of flavin monooxygenase-like enzymes—named *YUCCA* (*YUC*)—that catalyze the hydroxylation of the amino group of tryptamine, a rate-limiting step in IAOx tryptophan-dependent auxin biosynthesis pathway [91]. *YUC*s use NADPH and molecular oxygen to catalyze the oxidative decarboxylation of IPyA to IAA, using flavin as a cofactor [92,93]. Single *yucca* gene knockout mutants in *Arabidopsis thaliana* show wild-type growth phenotypes, likely because of redundancy among the 11 homologs [28]. Maize however possesses only 2 *yucca* copies and single mutants produce strong auxin-deficient phenotypes [94,95]. Overexpression of *YUCs* results in very limited effects on the IAA-levels, due to the strong homeostatic control of the active IAA-concentration but differences in IAA-metabolites can be clearly measured [87,93]. The *YUC* genes are mainly expressed in meristems, young primordia and reproductive organs, corresponding to sites of high auxin biosynthesis [69]. *YUCs* can also catalyze the decarboxylation of phenyl-pyruvate (PPA) to phenyl-acetic acid (PAA) [54]. The *TAA-* and *YUC-*family genes are regulated spatiotemporally and contribute to the uneven distribution of IAA in plants [25,26,66,67,96]. Homologs of the Arabidopsis *YUC* gene family have been found from several other plant species, suggesting a widespread occurrence of this branch throughout the plant kingdom.

Besides the linear pathway from IPyA to IAA, using the *YUC*s, indole-3-acetaldehyde (IAD) might be formed as an intermediate, like it happens in plant growth promoting rhizobacteria [97]. Whereas, the enzyme that could cause the conversion of IPyA to IAD has not been discovered in plants yet, the downstream aldehyde oxidases (AO) necessary to generate IAA out of IAD are present [98] (Blue Pathway in Figure 1).

### 2.2. The Indole-3-Acetamide (IAM) Pathway

IAM is the key intermediate in the bacterial auxin biosynthesis pathway of *Agrobacterium tumefaciens*. IAM is also found in many plant species including Arabidopsis, Citrus and Prunus, rice and maize [99,100,101]. By analogy with the bacterial synthesis route, the putative plant IAM-pathway would also encompass a two-step reaction with in the first step the conversion of Trp to IAM, which may be executed by a trypthophan-2-monooxygenase. Next, IAM is converted to IAA by the action of IAM HYDROLASE. Searching for orthologs of the bacterial *iaaH* in *Arabidopsis thaliana* has resulted in the identification of *AtAMI1* (*AMIDASE 1*) as a candidate plant IAM hydrolase [102] (Yellow Pathway in Figure 1). Also, *Nicotiana tabacum* harbors an *iaaH* orthologue, *NtAMI1*, which is required for growth of BY2 cultures on medium, in which NAA is depleted but indole-3-acetamide is supplemented as alternative auxin-source [103]. Homologs of *AMI* have been identified in other plant species including the monocot rice and algae, suggesting that this auxin biosynthesis route is evolutionary conserved [104,105,106]. *AMI1* homologs can convert phenyl-2-acetamide (PAM) into PAA, giving an explanation for the occurrence of auxinic molecules other than IAA in plants [107].

IAM may also be produced from indole-3-acetaldoxime (IAOx) because the IAM-levels are significantly decreased in mutants defective in the cytochrome P450 enzymes *CYP79B2* and *CYP79B3* (*cyp79b2cyp79b3*), involved in the IAOx pathway [98,108,109] (Yellow Pathway in Figure 1). However, in rice, maize or tobacco, species that lack the *CYP79B2*/3 homologs, IAM is still detected, which indicates that in these species the role of this pathway’s contribution to auxin biosynthesis is not entirely clear yet.

### 2.3. The Tryptamine (TAM) Pathway

Early reports of auxin activity in *Avena* curvature tests with tryptamine (TAM) suggest it acts as a precursor of auxin biosynthesis [105]. TAM is the first secondary metabolite derived from tryptophan in the synthesis of terpenoid indole alkaloids. In animals TAM is synthesized by tryptophan decarboxylase (TDC) in the cytosol [97] but a corresponding plant *TDC*-gene has hitherto not been found. TAM is converted to N-hydroxytryptamine in a rate-limiting step catalyzed by members of the *YUCCA* family [91] (Green Pathway in Figure 1). However, with the now completely described IPyA pathway, this double role of *YUCs* is questionable because N-hydroxytryptamine has not been detected in plants so far [89,93,110].

### 2.4. The Indole-3-Acetaldoxime (IAOx) Pathway 

The IAOx route is unique to the *Brassicaceae* family [101]. Two homologous cytochrome P450 enzymes, *CYP79B2* and *CYP79B3* catalyze the synthesis of IAOx from Trp. This step takes place in the chloroplasts [108,109] (Pink Pathway in Figure 1). In the *cyp79b2cyp79b3* double mutant, the IAOx levels were far below the detection limit, confirming the role of these enzymes [101]. In addition to its role as auxin precursor, IAOx is also shunted to several secondary metabolites such as indole glucosinolates (IG) and camalexin [111] (Pink Pathway in Figure 1). Mutations in glucosinolates biosynthesis genes *SUR1* (*superroot1*), *SUR2* or *UGT74B1* (*UDP-glucosyltransferase UGT74B1*) resulted in elevated auxin accumulation [112,113,114]. The blocked IG biosynthesis may cause an increased flux of IAOx to IAA [28]. Because the levels of indole-3-acetonitrile (IAN) were also decreased in the *cyp79b2cyp79b3* mutant, IAN is assumed to be a downstream intermediate of the IAOx pathway (Pink Pathway in Figure 1). Indeed, IAN can be formed by dehydration of IAOx and this reaction is catalyzed by CYP71A13 [115]. This IAN can be an intermediate for IAA but it is not found in all plant species [94]. Additionally, IAN is produced by hydrolysis of IG by myrosinases [116]. Auxin biosynthesis from IAN might involve nitrilases (NIT 1-4) but that process is not well understood yet [117,118] (Pink Pathway in Figure 1). The reduction of free IAA in the *cyp79b2cyp79b3* is more significant at high temperature. This could indicate that the IAOx-pathway in Arabidopsis is involved in temperature-induced or -regulated IAA-formation [109].

### 2.5. Tryptophan-Independent Auxin Biosynthesis Pathways

Because tryptophan synthase-deficient mutants of *Arabidopsis thaliana* showed higher levels of IAA-conjugates, it was suggested that Arabidopsis harbors a tryptophan-independent auxin biosynthesis route [78]. Indeed, the intermediates IGP and indole may condense to form IAA, without Trp as an intermediate [82,119] (Gray Pathway in Figure 1). Decorticating the Trp-independent auxin biosynthesis route awaits further research. For reviews on this pathway see [17,80,85].

## 3. Auxin Biosynthesis Regulates Root Embryogenesis

The initiation of the apical-basal body plan of a plant embryo occurs during the initial stages of embryogenesis and depends on auxin gradients, transport and local biosynthesis [120,121,122,123]. Manipulation of auxin homeostasis by conditional expression of *iaaM* (indoleacetic acid-tryptophan mono-oxygenase) or the expression of a *Pseudomonas syringae* indoleacetic acid-lysine synthetase (*iaaL*) did not interfere with embryo-patterning [124]. Additionally, the *sur* mutants produced normal embryos but displayed auxin-overproduction phenotypes after germination, which indicates that the IAOx-biosynthetic route is not active during embryogenesis [125]. These results suggest that during embryogenesis there is a compensatory mechanism for biosynthesis-mediated changes in auxin homeostasis. On the other hand, inhibition of transport by 1-N-Naphthylphthalamic acid (NPA), led to fused cotyledons [126], which indicates that proper auxin distribution is critical during embryogenesis. Mutant studies attributed a major role to the PIN1 and PIN4 auxin transporters to establish and maintain the auxin gradient in the embryo [124]. But because of the feedback regulatory role of auxin itself on PIN-trafficking and -localization also auxin biosynthesis must be tightly regulated during embryogenesis [127,128,129].

Initially, auxin is supplied by the maternal tissue but the mathematical models do not exclude that local auxin production takes place in the suspensor [129,130]. This theory is substantiated by specific expression of *YUC3*, *YUC4* and *YUC9* in the suspensor and the localization of PIN7 to direct the synthesized IAA towards the developing pro-embryo [122,131]. Afterwards, auxin production in the pro-embryo starts and *TAA1* expression is observed in the top apical cells of the 16-cell stage embryos. At a later stage, *YUC1* and *YUC4* are expressed in this region, while *YUC8* is expressed closer to the root pole [96,131,132]. While at the apical part of the developing embryo cotyledons are initiated, the hypophysis, the uppermost suspensor cell, develops into the root meristem with the stem cells and quiescent center (QC) and onset of radial tissue patterning necessary for post-embryonic root growth [3,14,133]. Mutants missing *YUC1*, *YUC4*, *YUC8*, *YUC10* or *YUC11* failed to develop a hypocotyl and a root meristem, which confirmed the role of auxin generated by the *YUCCA* flavin monooxygenases during embryogenesis [96]. The expression of the auxin biosynthesis genes (*TAA1/TAR* and/or different *YUC*) in different regions of the developing embryo is furthermore essential for vasculature development and the initiation of cotyledons [25,67]. A paralog of *Tryptophan synthase* α (*TSA*), *indole synthase* (*INS*) is thought to act in a pathway parallel to *TAA1*. Cellular divisions in the *ins-1* mutant embryo are disturbed and this indicates a role for *INS* during embryogenesis. Moreover, *INS* is expressed even earlier than *TSA* in the embryo and suspensor. Lastly, Trp-independent auxin biosynthesis is considered an important source of the plant growth regulator during embryo development [134].

## 4. Local Auxin Accumulation Regulates Root Development and Branching

A typical root system of dicotyledonous plants is composed of a primary root (PR) and lateral roots (LRs), which are secondary root branches, formed post- embryonically from PR to enable the plant to exploit the soil environment [135,136]. Common to all roots is the presence of a small group of cells at the center of the tip of the root organ. These cells are the stem cell niche, which coordinates the establishment of various tissues present in the root. Without these stem cells, root initials and the QC, the root can no longer grown and add cells to the developing tissue files. Critical to the activity of the QC and the root initials is the presence of an auxin maximum centered at the stem cell niche [62,137]. The formation of the auxin maximum at the root tip has been attributed to directional transport of shoot-derived auxin moving down into the root tip where complex patterns of auxin efflux carriers result in auxin accumulation at the stem cell niche [6,138,139,140]. Maintenance of the auxin maximum is however not solely the result of transport but also involves local auxin biosynthesis [141], which is possible in all parts of a root system [62,69]. Genes involved in Trp production (*ASA1*) and auxin biosynthesis genes encoding proteins of the IPyA pathway (*TAA1, TAR2* and *YUCCA*) are expressed in the root tip [25,67,142]. The expression of *YUC* genes at the root tip depends on symplastic communication between the cells of the stem cell niche, suggesting that stem cell maintenance is intrinsically linked with the maintenance of a local auxin maximum [141]. Auxin derived from the shoot does not fully rescue the root growth and gravitropic responses characteristic for auxin-deficiency at the root tip, emphasizing the importance of local synthesis for root development [142]. In addition, locally synthesized auxin has been shown to rescue a local phenotype, yet does not show effects at a distance [66,96,143]. Also, root growth and patterning is strongly affected by deficiencies in local IPyA-mediated auxin biosynthesis. In rice, disruption of the *FISH BONE* gene, an orthologue of *TAA1*, affects many auxin-mediated processes including root development [144]. Antisense expression of *YUCCA1* (*OsYUC1*) in rice results in a defective root, which phenocopies the root of auxin-insensitive mutants [145]. Similarly, loss of function mutants of the rice *OsTDD1* gene, encoding anthranilate synthase β-subunit, have severe root defects but their phenotype can be rescued by overexpression of *OsYUC1* [146]. In woodland strawberry (*Fragaria vesca* L.), silencing of *YUCCA6* affected various post-embryonic organ developmental steps including root formation [147]. Many steps of Trp-synthesis involve transamination reactions, which require vitamin B6 as a cofactor. The *pdx1* mutant, a vitamin B6 biosynthesis mutant with a short root, is defective in the generation of IAA from tryptophan. The root defects in this mutant are another indication that PR growth depends on Trp-dependent local auxin biosynthesis [148]. Other auxin biosynthetic pathways, such as the IAOx pathway are involved in root growth as well. For example, *AtTGG4* and *AtTGG5* act during the conversion of indole glucosinolates (IG) to the auxin precursor, indole-3-acetonitrile (IAN) and show defects in the establishment of an auxin gradient in the root tips resulting in a disturbed root growth [116]. Downstream nitrilase enzymes, such as *NIT1*, are involved in auxin biosynthesis in the root [149]. Furthermore, simultaneous inactivation of *cyp79b2* and *cyp79b3* results in a significant reduction of the auxin levels at the root tips [69,109]. However, due to redundancy in the different auxin biosynthetic pathways, most of the single biosynthetic mutations did not show dramatic root defects.

LRs develop from pericycle cells, adjacent to the xylem poles [150]. Within these so-called xylem pole pericycle founder cells, cell cycle reactivation and subsequent divisions result in the formation of an LR primordium. Priming of the founder cells occurs in the basal root meristem where it requires oscillating auxin fluxes [151,152,153,154]. The generation of IAA out of IBA seems to play a critical role in these oscillation patterns [58,59]. Programmed cell death at the lateral root cap is a second cause for the oscillatory auxin responses because it releases pulses of auxin into the surrounding root tissues [155]. Upon priming, the pericycle cells proceed through two cycles of asymmetric divisions, which gives rise to the formation of a single layer lateral root primordium (LRP) [156]. Further periclinal divisions yield a dome-shaped multilayer LRP, ready to emerge from the main PR [157,158,159]. Without the endogenous priming in the root meristem, a random stimulation of auxin biosynthesis in a pericycle cell results in an increase of auxin, sufficient to start the formation of an LR [160]. The Arabidopsis *superroot1 (sur1)* and *sur2* have excessive amounts of LRs, indicating that local accumulation of auxin is sufficient to induce LR and AR formation [98,112,161,162]. These findings indicate that auxin accumulation is a trigger to modify the developmental program of the pericycle cell [163]. In young seedlings, early after germination, LR emergence depends on shoot-derived auxin but the capacity to synthesize auxin increases with the age of seedlings, which in turn reduces the dependence on shoot-derived auxin for LR development and local auxin biosynthesis takes over to control LR emergence [70]. The local synthesis of auxin appears to depend on auxin transport because disruption of the GNOM-dependent auxin transport resulted in reduced expression of *YUCCA* genes (*YUC2, YUC3, YUC5* and *YUC6*) and *TAA*1 and *TAR2*. In gnom this reduced auxin accumulation at the sites of LRP formation resulted in fewer LR. A complex regulatory network combining auxin transport and local biosynthesis hence plays an important role in the initiation and further development of LR [164].

## 5. Importance of Auxin Biosynthesis for Adventitious Root Development

Adventitious root (AR) emerge from aerial parts of the plant in response to wounding, flooding and other stresses, or as part of a root developmental program [165,166,167]. There are two types of ARs: roots originating from latent and ready-made initials and de novo initiated AR [168]. Generally, ARs are initiated from tissues close to the vascular tissues including the pericycle in the hypocotyl, phloem or xylem parenchyma cells, interfascicular cambium cells, procambium and vascular parenchyma cells [10,11,168,169].

Like many other developmental processes, AR formation is regulated by external cues such as light, temperature and nutrient availability, which generate endogenous hormone signaling events [168,170]. However, wounding is probably the most powerful signal that induces de novo rooting process, especially when the shoot organ is severed from the root. A primary response to wounding is the accumulation of auxin by means of directional auxin transport and local auxin biosynthesis. In petunia cuttings, IAA peaked at the base of the stem early post-excision accompanied by a fast induction of *YUCCA*-family members and two IAA-amino acid hydrolases [171]. Their combined activity leads to a higher IAA-concentration typical for a wound-response [172]. A similar study in *Arabidopsis thaliana* showed that following root pruning the endogenous auxin levels rise due to polar auxin transport and increased expression of *YUCCA9* [173,174]. Also for AR induction on detached leaves, *YUCCA-*mediated auxin synthesis was associated with root initiation [174]. For wound responses, *YUC1* and *YUC4* play the most important roles but it is not known yet how wound signals trigger these biosynthetic genes [174,175]. *TAA1* family members are not induced by wounding, yet mutants lacking *TAA1* and its closest homologue *TAR2* are required for formation of roots on detached leaves [176]. *YUC6*-mediated auxin biosynthesis is needed at the tip of the AR to stimulate post-emergence growth, similar to local auxin production in the PR and LR tips [62,177,178]. This local auxin biosynthesis compensates for the reduction of auxin flow to the tip in the presence of cytokinin [177].

In general, easier-to-root varieties contain higher levels of free auxin whereas rooting-recalcitrant varieties have less auxin [179]. The positive correlation between auxin-levels and AR induction is further substantiated by spontaneous AR formation in auxin-overproduction mutants. Transgenic rice overexpressing *YUCCA* develop more crown roots [145]. The *superroot* mutants *sur1* and *sur2* are blocked in glucosinolated indole synthesis and thereby re-channel IAOx for IAA-synthesis, resulting in excessive AR formation [108,112,113,161,162]. The spontaneous rooting in *sur1* and *sur2* is lost in mutants with reduced Trp synthesis (e.g. mutants in *ASA1, ASB1* and *TSB1*). However, other suppressors of *sur2* are not controlling endogenous IAA-levels but are affected in auxin signaling [168]. A high auxin concentration in the plant is therefore as such not sufficient to induce AR.

## 6. Regulation of Auxin Homeostasis during Root Development by Other Hormones

The balance between auxin and cytokinin is of critical importance for determining the developmental fate of organs, with auxin promoting the root and cytokinin stimulating shoot development. At intermediate auxin/cytokinin ratios, callus, predominantly existing of actively dividing cells, is induced reflecting the stimulatory activity of both auxin and cytokinin [177,180]. The strong link between auxin and cytokinin (co)-activity is mediated by regulatory circuits that control each other’s production and transport. Constitutive downregulation of cytokinin was reflected in a significant downregulation of auxin biosynthesis [178]. Furthermore, cytokinin strongly inhibits PIN-type auxin efflux carriers [181,182,183]. On the other hand, cytokinin-induced repression of the auxin transport machinery resulted in secondary stimulation of the auxin biosynthetic gene *YUCCA6* [177,184]. The connectivity between auxin and cytokinin is not necessarily conserved across plant species but in for example poplar and Arabidopsis, auxin biosynthesis was induced by an increase in cytokinin [185,186]. Microarrays and q-RT-PCRs showed altered expression of metabolic genes from different auxin biosynthetic pathways upon cytokinin treatment, confirming that cytokinin interferes with auxin biosynthesis [187]. One of these genes is the *Cytokinin-induced Root Curling 6 (CKRC6)*, which is allelic to *ASA1.* The *ckrc6* mutant is insensitive to both cytokinin and ethylene while containing less IAA [188]. This results in a reduced gravitropic root growth phenotype, which can be compensated by exogenous application of auxin. The downstream Trp synthesis gene *ASB1* is also regulated by cytokinin by means of the response regulator *ARABIDOPSIS RESPONSE REGULATOR* (*ARR1*). Cytokinin-treatment of the root system leads to an increase in auxin at the transition zone of the root meristem due to locally increased *ASB1* expression [189].

The cross-talk between ethylene and auxin inhibits root elongation and regulates LR initiation and emergence [190,191]. Ethylene regulates the expression of two *WEAK ETHYLENE INSENSITIVE* (*WEI2/ASA1* and *WEI7/ASB1*) genes, which encode subunits of anthranilate synthase, a rate-limiting enzyme in Trp biosynthesis [25,192]. These genes were identified in a screen for ethylene insensitivity. *WEI2* is allelic to *ASA1*, involved in Trp biosynthesis and *WEI8* to *TAA1*, an enzyme in the IPyA auxin biosynthetic pathway. These mutants underscore the link between ethylene signaling and auxin biosynthesis [25]. In ethylene-treated seedlings, an overall increase of the auxin response at the root tip was observed and this is also reflected in direct auxin measurements [191,193]. A member of ethylene-responsive AP2 transcription factors; *ETHYLENE RESPONSE FACTOR1* (*ERF1*) was recently shown to bind the promoter of *ASA1* in order to regulate auxin biosynthesis and ethylene-induced root growth inhibition [194]. Another interaction between ethylene and auxin biosynthesis was discovered in a chemical genetic strategy, using L-kynurenine, a chemical that represses the nuclear accumulation of the *ethylene insensitive 3 (EIN3)* transcription factor and *TAA1/TAR* were identified as a target for L-kyn [195]. Lastly, rice transgenic plants overexpressing *OsEIL1*, the closest homolog of *EIN3*, exhibited a short, coiled primary root and they had increased *YUC8/REIN7* expression so auxin biosynthesis through the IPyA pathway is activated [196].

Root growth is also affected by the interplay between auxin and jasmonic acid (JA). Exogenous application of methyl jasmonate enhances LR formation but inhibits PR elongation [197,198]. Jasmonates reduce LR formation in the Arabidopsis *jasmonate-induced defective lateral root1* (*jdl1/asa1-1*) mutant. Inactivation of *JDL1/ASA1* suppressed LR formation and reduced auxin accumulation in the basal meristem, which indicates that jasmonates not only affect auxin biosynthesis but also affect auxin transport [199]. The molecular mechanisms modulating the crosstalk between jasmonate and auxin biosynthesis during LR formation act through the *Ethylene responsive transcription factor 109 (ERF109)*, which binds specifically to the promoter elements of *YUC2* and *ASA1*. The JA-signaling pathway is also linked to auxin homeostasis through the modulation of *YUCCA8* and *YUCCA9* expression [200]. In conclusion, the auxin gradients needed for root development, secondary root initiation and emergence depend on synchronized interactions between auxin biosynthesis and interaction with other phytohormones.

## 7. Auxin Biosynthesis as an Integrator of Environmental Factors and Root Development

The primary function of the root system is to search for water and minerals and to provide physical support for the shoot to grow towards a light source. Hence light, water, minerals and gravitropic force influence root development and architecture. Light has been reported to regulate the endogenous level of auxins [201,202]. During AR formation, light influences the expression of the auxin responsive *GH3* and *ARF* genes [203,204,205,206]. In *Arabidopsis thaliana*, the photomorphogenesis mutant *red1 defective in SUR2/ATR4 (ALTERED TRYPTOPHAN REGULATION 4*) failed to produce AR on the hypocotyl in red light [207]. Light is also shown to induce *YUCCAs* in the maize root [208]. De novo root organogenesis in Arabidopsis leaf explants involves different *YUCCA* genes depending on whether explants are incubated in the light or dark. The relevance of light as a factor regulating auxin homeostasis also follows from the identification of shade avoidance mutants some of which were affected in auxin biosynthesis genes. Tao et al. [67] identified *TRYPTOPHAN AMINOTRANSFERASE OF ARABIDOPSIS* (*TAA1*) in a shade avoidance mutant screen. TAA1 indeed contributes to local auxin biosynthesis during the elongation of the hypocotyl [209]. In low R:FR conditions, PHYTOCHROME INTERACTING FACTOR 7 (PIF7) is activated via dephosphorylation and binds to the *YUCCA* genes to regulate their expression [210]. This suggests a direct link between the light-mediated PHYTOCHROME B (PHYB) pathway and auxin accumulation.

In Arabidopsis seedlings, exogenous application of sugar enhanced the PR growth and increased LR density by activating auxin biosynthesis [211]. The enhanced root growth correlated with transcriptional activation of the auxin biosynthesis genes *YUC2*, *YUC8*, *YUC9*, *CYP79B2*, *CYP79B3*, transporters *PIN1*, *PIN2* and signaling *AUX/IAA* genes [211,212]. Similarly, sucrose grown seedlings develop elongated roots and auxin accumulates in the shoots and roots [213].

Root branching is enhanced in nutrient rich-soils [214,215,216,217]. Nitrate is a growth-limiting nutrient and the major source of nitrogen (N) for plants [218]. High and low concentrations of N significantly inhibit respectively enhance LR development with no or limited effect on growth of the PR [219,220]. N-depletion suppresses auxin-levels in the shoot while it stimulates auxin accumulation in the root, in line with a reduction in *TAA1* and *TAR1* expression in the shoot and upregulation in the root [221,222,223]. Also in wheat, *TAR* transcription is affected by N-supply [224]. Not much is known about the mechanism by which N-availability regulates auxin biosynthesis genes. The overexpression of *AGAMOUS-like 21 (AGL 21)* has been shown to stimulate LR elongation as a function of *N*-availability in agreement with enhanced expression of *YUC5, YUC8* and *TAR3* [225]. An *agl21* mutant had fewer and shorter LRs and exogenous auxin application rescued the root phenotype, pointing to a role in the regulation of auxin biosynthesis. In Arabidopsis, at low concentrations of phosphate, PR growth is impaired, while LR development and root hair formation are promoted [226]. The working hypothesis is that auxin accumulation is required to ameliorate root growth under phosphate-starving conditions and overexpression of *YUC1* promotes root architecture changes in P-limiting conditions [227]. *NFYA* (*NUCLEAR FACTOR Y A*)-B1 responds to nitrate- and phosphate-starvation by modulating the expression of *TAR2* [228]. Finally, sulphate-availability modulates transcriptional activation of *nitrilase 3 (NIT3)* and this leads to enhanced local auxin biosynthesis [229].

Gravitropism ensures roots to grow downward in their search of sufficient nutrients and water [230]. In the Arabidopsis root, an asymmetric auxin gradient between the lower and upper sides of roots is responsible for root curving in response to gravity [231,232]. Besides a differential auxin transport, differential *TAA1* activation in the lower and upper epidermal cells, aids in the formation of an asymmetric auxin gradient and response to the gravity vector [26].

The PR elongates under mild drought conditions, allowing access to deeper water reserves and this growth is supported by modulating auxin activity in the PR meristem. Transgenic plants (Arabidopsis, potato and poplar) overexpressing *iaaM*, *YUC6* or *YUC7*, produce more IAA and show enhanced drought resistance, while the *yuc1/yuc2/yuc6* triple mutant is more sensitive to drought stress [233,234,235,236,237]. However, co-overexpression of *iaaL*, an IAA-conjugating enzyme and *YUC6*, reduces the endogenous IAA-levels but drought stress tolerance remained unaffected, which indicated that the tolerance is not solely based on IAA-overproduction or -levels [233]. Also, *IAA-Ala Resistant3 (IAR3)* plays a role in modulating root architecture during osmotic stress. This hydrolase is capable of generating free IAA from IAA-amino acid conjugates under drought stress. The locally generated auxin then stimulates LR development to promote plant survival in dry conditions [238]. Water availability stimulates auxin biosynthesis and -transport via the regulation of *TAA1* and *PIN3*. Newly initiated LR are preferentially initiated towards the water gradient and this as well might be mediated by local auxin-biosynthesis [239]. This hydro-patterning is reduced in *wei8* and *sav3 but* can be rescued by constitutive expression of *TAA1* in the root epidermis [240].

Aluminum (Al), when present as Al^3+^ ion, constitutes a major treat to the survival of plants in acidic soils (pH < 5) [241,242]. Al-induced inhibition of root growth is mediated by auxin [243,244,245]. In Arabidopsis, Al induced *TAA1* directly or through the intervention of ethylene and as a consequence inhibited root growth [245,246]. Similarly, auxin-overproducing *yucca* mutants had an increased Al-sensitivity and contributed to the root growth inhibition in response to Al-stress [247,248]. Besides an effect on auxin biosynthesis, a reduction of *PIN2* expression and localization contributed to the Al-induced root inhibition [249,250]. The metal ion of manganese (Mn) also inhibited PR growth and LR development by reducing the expression of *YUC2, YUC3, SUR1, ASA1, PIN4* and *PIN7* [251]. Boron-deprivation led to a significant increase of the endogenous auxin content thanks to the induction of *TAA1*, *TAR2*, *YUC3* and *YUC8* together with a decrease of the rootward IAA-transport (mediated by PIN1 and PIN4) [252].

In addition to abiotic environmental factors, root growth is influenced by beneficial growth-promoting rhizobacteria (PGPR) or fungi (PGPF) growing in the vicinity, on or in root tissue and pathogens [253]. The growth hormone auxin plays an important role in the communication between these microbes and the plant. Many plant-pathogenic microorganisms produce indole-3-acetic acid (IAA), which often determines their pathogenicity or influence endogenous auxin biosynthesis [254,255,256]. In general, both abiotic and biotic stresses and environmental conditions affect root development through the modulation of auxin biosynthesis.

## 8. Meta- and Catabolic Processes Controlling Auxin Levels

For auxin to display its crucial role during plant development, the endogenous concentration has to be spatio-temporally regulated. The role of biosynthesis has been discussed above but also inactivation, metabolism and storage contribute to the establishment of local levels of active auxin. IAA is known to act as a free acid but also conjugated forms, with a wide variety of sugars, peptides and amino acids are found in plants [17,18,65,257].

Conjugation of IAA occurs predominantly at the carboxyl group involving amino acids, sugars and other primary metabolites in order to render IAA inactive [19]. Conjugation could reversibly modulate free IAA levels and hence allows rapid and versatile responses to environmental changes and developmental [258,259,260]. IAA–amino acid conjugates can be classified into two groups based on in vitro activity and *in planta* feeding assays. Conjugation of IAA to aspartate (Asp), cysteine (Cys), histidine (His), isoleucine (Ile), lysine (Lys), proline (Pro), tryptophan (Trp) and valine (Val), various alcohols and sugars, serves as a way to inactivate IAA and to label it for degradation [255,256,258,259,260], whereas conjugation to alanine (Ala), leucine (Leu), phenylalanine (Phe), asparagine (Asn), glutamine-(Gln), glutamic acid (Glu), glycine (Gly), methionine (Met), serine (Ser), threonine (Thr) and tyrosine (Tyr), myo-inositol, or peptides creates temporary storage forms that can generate free IAA by hydrolysis [21,259,260,261,262,263]. Not only exogenous 2,4-d but also its amide-linked metabolite 2,4-d-Glu displayed an inhibitory effect on plant growth via the *TIR1*/*AFB* auxin-mediated signaling pathway and this metabolite can be found endogenously in the 2,4-d-treated plants [264]. At least seven members of the GRETCHEN HAGEN 3 (GH3) protein family have been shown to be involved in IAA-conjugation to amino acids and their expression is regulated by free auxin [20,265] (Purple pathway Figure 1). The development of adenosine-5′ (2-(1*H*-indol-3-yl) ethyl) phosphate (AIEP), a small molecule inhibitor of IAA-amido synthase, has helped the functional analysis of IAA-amido synthases [266]. The hydrolysis of IAA-conjugates to free IAA is regulated by IAA-LEUCINE RESISTANT 1 (*ILR1*), IAA-LEUCINE RESISTANT-LIKE 2 (*ILL2*) and IAA-ALANINE RESISTANT 3 (*IAR3*) [257,267,268,269] (Purple pathway Figure 1). The inactive IAA-methyl ester, Me-IAA, is generated by *IAA CAROXYMETHYLTRANSFERASE1 (IAMT1)* and hydrolyzed by *METHYL ESTERASE17 (MES17)* (Purple pathway Figure 1). Me-IAA has been shown to play a role in gravitropism, it exerts, like IAA, an inhibitory activity on root and hypocotyl growth, leaf development and is involved in plant fertility because RNAi IAMT1 plants form less seeds [270,271]. However, MeIAA does not function by itself but exogenous application results in *in planta* hydrolysis and release of free IAA. Interestingly, blocking the conversion of auxin storage forms to free IAA results in a compensating activity of the IPyA biosynthetic pathway [272].

IBA can be considered a precursor of IAA. IBA is metabolized slower than other conjugates and less prone to non-enzymatic degradation. Furthermore, IBA does not undergo oxidation [258]. IBA is transported in the root, probably by the carrier proteins *PLEIOTROPIC DRUG RESISTANCE 8 (PDR8)* and *PDR9* [6,58]. IBA-activity depends on peroxisomal import through *PEROXISOMAL ABC TRANSPORTER1 (PXA1)* and subsequent β-oxidation to IAA. The peroxisomal enzyme 3-ketoacyl-CoA thiolase PED, contributes to both fatty acid and IBA β-oxidation. Despite ample reports on bioactivity of IBA, its biological role is still controversial [273,274] (Purple pathway Figure 1).

The inactivation of auxin by oxidation resulting in the formation of the catabolite 2-oxindole-3-acetic acid (oxIAA) has only recently been studied in more detail but was already longtime considered a main auxin catabolism pathway [275,276]. oxIAA is an irreversible IAA catabolite with little bio-activity and it is not transported via the canonical polar auxin transport system [277]. Recently, three groups independently published back to back on the role of the Arabidopsis gene *DIOXYGENASE FOR AUXIN OXIDATION 1* (*AtDAO1*) to catalyze the formation of oxIAA both in vitro and in vivo [278,279,280] (Purple pathway Figure 1). *DAO* was previously characterized in rice to have a role in auxin metabolism and reproductive development [281]. Arabidopsis mutants lacking this enzyme contain 50–95% less oxIAA (resp. data from [20,279]) and have phenotypes reminiscent of elevated auxin levels [279,280]. A second isoform, *AtDAO2*, is only expressed in the root cap and is also involved in the formation of oxIAA. Both genes are closely related to the apple *Adventitious Rooting Related Oxygenase* 1 (*ARRO1*) and rice *DAO* [280,282,283]. Temporal and tissue-specific inactivation of auxin by *AtDAO1* is essential to regulate the plant’s endogenous auxin levels. The localization of *AtDAO1* in the border cells flanking the lateral root primordia (LRP), suggests that this enzyme is important to create an auxin maximum at the center of the root primordium [279]. In mutants lacking *AtDAO1*, the expression of *GH3s* as well as the synthesis of IAA-Asp and IAA-Glu is upregulated. This suggests that there is a compensation feedback between the different redundant IAA-regulating pathways [20,279,280]. In plants, there is a fast auxin conjugation response through the formation of conjugates such as IAA-Asp, which is more active at higher auxin concentrations and a constitutive slow conjugation response through oxidation and oxIAA formation, which acts at lower auxin concentrations [20,279]. The increase in auxin production in the *dao1* mutant is mainly mediated via the IPyA pathway [20,280]. *AtDAO2* was shown to follow a circadian rhythm-based oscillation in the root cap, which is a trigger for the activation of lateral root initials [58,59,281].

The generation of oxIAA is followed by a subsequent glycosylation to oxIAA-glucoside (oxIAA-Glc), which is very efficiently executed by UGT74D1 [284] (Purple pathway Figure 1). UDP-glycosyltransferases (UGTs) are one of the largest families of glycosyltransferases in plants [281,283] and some of them act in the conversion of IAA to glucose-conjugates. UGT84B1 catalyzes the conversion of IAA to 1-*O*-(indol-3-ylacetyl)-β-d-glucose (IAA-Glc), while UGT74E2 catalyzes the formation of 1-*O*-(indol-3-ylbutanoyl)-β-d-glucose (IBA-Glc) from indole-3-butylic acid (IBA) and UGT74D1 converts both IAA and IBA but also NAA, 2,4-D and ICA to their corresponding glucosides [285,286,287]. The *iaglu* gene encodes IAA glucosyltransferase in *Zea mays* [288]. In Arabidopsis over 100 uridine diphosphate (UDP)-glycosyltransferases (UGTs) have been identified that are classified into 14 subgroups [289,290]. Members of the L subgroup are potentially involved in the formation of IAA-glucose esters. Overexpression of *UGT74D1* results in the loss of root gravitropism presumably due to increased glycosylation of ox-IAA and reduced IAA level at the root tip [284]. At the whole plant level, no decrease in IAA was observed, which suggest a compensating feedback mechanism [284]. A similar phenomenon was observed in plants overexpressing *UGT84B1* in which an increase of IAA was measured [291]. The overexpression of *UGT74E2* on the other hand led to an increase in IBA [287]. To allow for a temporal- and concentration-dependent regulation of auxin homeostasis, it was shown that *GH3s* act fast while *UGT74D1* was slower upon auxin treatment. These results suggest that the OxIAA and *GH3*-pathways have distinct roles in IAA-homeostasis. The OxIAA pathway may function constitutively to maintain the basal levels of IAA in plants while the *GH3* pathway may play a role in cases where plant cells have to rapidly reduce the relative amount of IAA in response to developmental and environmental changes [284].

Last but not lease, conjugation compartmentalization of IAA in the endoplasmic reticulum has been shown to regulate the availability of IAA to cellular conjugation mechanisms [292].

## 9. Conclusions

What have we learned so far from auxin-related studies is that the combined action of auxin biosynthesis and transport set the stage for adaptive developmental processes. The root system in particular is sensitive to auxin. Whereas the last decade’s emphasis was more on auxin redistribution by means of polar and local transport, current research progress underscores the importance of local auxin biosynthesis. Auxin biosynthesis genes have been found to play a role in every aspect of root development translating environmental cues into adaptive responses (see Table 1). As auxin, derived from distantly located apical origins as well as locally synthesized auxin equally have impact on root architecture and development, the mechanism by which auxin is perceived and possibly how it enters the cells may hold specific information relevant for the adaptive growth responses. Future research should focus on local auxin signaling and spatial and temporal perception mechanisms controlling auxin-mediated establishment of root meristems.

## Figures and Tables

**Table 1:** Summary auxin biosynthetic genes in root development.

Biosynthetic Pathway	Biosynthetic Pathway	Biosynthetic Pathway	Biosynthetic Pathway
*iaaM*	Pseudomonas/Agrobacterium tryptophan-2-monooxygenase	Auxin biosynthesis in transgenic overexpression lines	Trp → IAM
*iaaH*	Pseudomonas/Agrobacterium IAM hydrolase	Auxin biosynthesis in transgenic overexpression lines	IAM → IAA
*IPDC*	IPA decarboxylase	Bacterial pathway	IPyA synthesis
*ASA1/WEI2/JDL1/CKRC6/ASA2* *ASB1/WEI7*	Subunits of anthranilate synthase	Root tip expression, inhibitor of *sur1* Hormone interactions (cytokinin/ethylene/jasmonic acid)	Chorismate → anthranilate
*IGS*	Indole-3-glycerol phosphate synthase	Catalyzes formation of key intermediate of Trp-dependent and Trp-independent pathways	Anthranilate → indole-3-glycerol phosphate
*TSA1/TSB1-2*	Trp synthase a and Trp synthase b	Trp synthesis	Indole-3-glycerol phosphate → indole → Trp
*INS*	TSA homolog	Early expression in embryo	
*PDX1*	vitamin B6 biosynthesis mutant		Trp → IAA
*TAA1/TAR1-4*	Arabidopsis tryptophan aminotransferases	Stem cell niche specification *De novo* root organogenesis Control root architecture nitrogen dependently	Trp → IPyA
*TAAI/WEI8/SAV3/TIR2*	Arabidopsis		Trp → IPyA
*TaTAR2*	wheat	Lateral root formation Nitrate and phosphate starvation	IPyA
*FISHBONE*	Rice Orthologue TAA1		
*OsYUC1* *OsCOW1*	Rice Flavin-containing monooxygenases	Post embryonic root development Crown and adventitious roots	IPyA
*YUC1-11*	Arabidopsis Flavin-containing monooxygenases	Root embryogenesis RAM maintenance Drought resistance *De novo* root organogenesis Root growth in Al^3+^ stress	IPyA → IAA TAM → *N*-hydroxytryptamine?
*YUC (ZM2G141383)*	Maize Flavin-containing monooxygenases	Root cap-mediated gravitropic U-turn in response to light	IPyA → IAA
*MYROSINASE* *AtTGG4/AtTGG5*	Arabidopsis	Local auxin distribution in the root	IG → IAN
*Nitrilases NIT1-4*	Arabidopsis	Root development and growth	IAN → IAA
*CYP79B2/CYP79B3*	Arabidopsis Cytochrome P450	Auxin gradients in the root	Trp → IAOx
*IBR1,3,10/ECH2*	INDOLE- 3-BUTYRIC ACID RESPONSE (IBR) ENOYL–COA HYDRATASE2	IBA to IAA conversion Priming LR founder cells	IBA → IAA
*AO*	Aldehyde oxidase	IPyA to IAD pathway?	IDA → IAA
*AMI1*	IAM HYDROLASE orthologue of *iaaH*	Required for BY2 growth on IAM medium	IAM → IAA PAM → PAA
*SUR1/SUR2*	Glucosinolate biosynthesis genes SUPERROOT1/2	Overproduction LR and AR	IAOx → IG
*UGT74B1*	UDP-glycosyltransferase	High auxin levels in mutant Mutation in IG synthesis	
*CYP71A13*			IAOx → IAN
*ILR1/ILL2/IAR3*	IAA-leucine resistant1 IAA-leucine resistant-like 2 IAA-Ala Resistant3 hydrolase		Conjugated IAA → IAA
*GH3*	GRETCHEN HAGEN 3 (*GH3*) protein family	IAA-conjugation to amino acids	IAA → amide conjugated IAA
*DAO*	*DIOXYGENASE FOR AUXIN OXIDATION 1*		IAA → oxIAA

## Abbreviations

2,4-d	2,4-dichlorophenoxy acetic acid
4-Cl-IAA	4-chloroindole-3-acetic acid
ABP1	auxin binding protein 1
*AFB*	*AUXIN SIGNALING F-BOX (AFB)* family of auxin receptors
AO	aldehyde oxidases
AR(s)	adventitious root(s)
*ARF*	*auxin response factor*
Aux/IAA	AUXIN/INDOLE-3-ACETIC ACID proteins
IAA	indole-3-acetic acid
IAD	indole-3-acetaldehyde
IAM	indole-3-acetamide
IAN	indole-3-acetonitrile
IAOx	indole-3-acetaldoxime
IBA	indole-3-butyric acid
IG	indole glucosinolates
IGS	indole-3-glycerol phosphate synthase
IPyA	indole-3-pyruvic acid
LR(s)	lateral root(s)
NAA	1-naphthaleneacetic acid
PAA	phenyl acetic acid
PIN	PIN-formed family of auxin transporters
PHYB	PHYTOCHROME B
PR	primary root
QC	quiescent center
RAM	root apical meristem
SAM	shoot apical meristem
SCF	ubiquitin protein ligase complex, with subunits: Skp1, Cullin and F-box
*TAA1/TAR1-4*	*TRYPTOPHAN AMINOTRANSFERASE OF ARABIDOPSIS 1/TRYPTOPHAN AMINOTRANSFERASE RELATED 1-4*
TAM	Tryptamine
*TIR1/2*	*TRANSPORT INHIBITOR RESPONSE ½*
Trp	Tryptophan
TSA1	Trp synthase a
TSB	Trp synthase b
*YUC1-11*	*yucca* genes encoding flavin-containing monooxygenases
